# Non-Darcy interfacial dynamics of air-water two-phase flow in rough fractures under drainage conditions

**DOI:** 10.1038/s41598-017-04819-x

**Published:** 2017-07-04

**Authors:** Chun Chang, Yang Ju, Heping Xie, Quanlin Zhou, Feng Gao

**Affiliations:** 1 State Key Laboratory of Coal Resources and Safe Mining, China University of Mining & Technology at Beijing, D11 Xueyuan Road, Beijing, 100083 P. R. China; 2 State Key Laboratory for Geomechanics and Deep Underground Engineering, China University of Mining & Technology, No 1, University Avenue, Xuzhou, 221006 P. R. China; 3 Key Laboratory of Energy Engineering Safety and Mechanics on Disasters, The Ministry of Education, Sichuan University, Chengdu, 610065 China; 4 Energy Geosciences Division, Lawrence Berkeley National Laboratory, Berkeley, California CA94720 USA

## Abstract

Two-phase flow interfacial dynamics in rough fractures is fundamental to understanding fluid transport in fractured media. The Haines jump of non-Darcy flow in porous media has been investigated at pore scales, but its fundamental processes in rough fractures remain unclear. In this study, the micron-scale Haines jump of the air-water interface in rough fractures was investigated under drainage conditions, with the air-water interface tracked using dyed water and an imaging system. The results indicate that the interfacial velocities represent significant Haines jumps when the meniscus passes from a narrow “throat” to a wide “body”, with jump velocities as high as five times the bulk drainage velocity. Locally, each velocity jump corresponds to a fracture aperture variation; statistically, the velocity variations follow an exponential function of the aperture variations at a length scale of ~100 µm to ~100 mm. This spatial-scale-invariant correlation may indicate that the high-speed local velocities during the Haines jump would not average out spatially for a bulk system. The results may help in understanding the origin of interface instabilities and the resulting non-uniform phase distribution, as well as the micron-scale essence of the spatial and temporal instability of two-phase flow in fractured media at the macroscopic scale.

## Introduction

Two-phase fluid flow and transport in naturally and artificially fractured media are of interest for many engineering applications, such as (1) tight reservoir exploitation for oil and methane recovery with environmentally conscious hydraulic fracturing in recent decades^1, 2^; (2) the geological sequestration and leakage evaluation of nuclear waste and anthropogenic CO_2_^3, 4^; (3) geotechnical applications for associated engineering disasters, including tunnel deformation and coal-water/gas outbursts during deep mining^5, 6^; and (4) geothermal and enhanced geothermal systems^7^. The spatially varying multiscale heterogeneity in fractured media makes two-phase flow modelling and prediction challenging because of the complex aperture variabilities^8–10^, fracture propagation and intersection^11, 12^. Recent laboratory experiments have shown significant dynamic and non-equilibrium behaviour for two-phase flow and mass transfer at multiscale in heterogeneous porous media^13–16^. However, the fundamentals of flow dynamics in fractured media, especially in single rough fractures that form the basic element of fractured media, remain poorly understood.

One of the challenges encountered to date is accurate quantification and characterization of fracture characteristics^8, 17^. The classical model considering a fracture bounded by a pair of smooth, parallel plates is no longer valid for fluid flow modelling^18–27^. The fractal dimension method (FDM) is an efficient characterization technique to account for the spatial variability of fracture aperture (or irregular fracture-surface roughness), with widely exhibited self-affine fractal properties of natural fractures regardless of the host rock type, the fracture direction, and the fracture formation mechanism^28–32^. The key parameter fractal dimension (*D*) is defined by a ratio providing a statistical index of complexity comparing how detail in a pattern (strictly speaking, a fractal pattern) changes with the scale at which it is measured. For natural fracture surface in two dimensions, *D* tends to fall approximately in the rage 2 ≤ *D* ≤ 2.5, while for the one-dimensional surface profile, it was measured in the range of 1.0 ≤ *D* ≤ 1.6^29^. Some studies of fluid flow and transport in fractures with FDM-fractal aperture have been reported^33–35^. In a two-dimensional rough fracture surface, experimental results from Ishibashi *et al*.^35^ show the channelling fluid flow within 1–2 narrow paths while the majority of the surface domain is bypassed, indicating the significant effect of single one-dimensional surface profile. At ~100 mm length scale, deviations of air-water interface velocity from experiments vs. LBM modelling in the rough fracture (i.e., *D* = 1.5) were observed by Ju *et al*.^36^, concluding that further detailed investigations on the mechanisms of two-phase flow and interfacial dynamics are required.

The two-phase interface displacement and interactions with the solid surfaces of complex geometry greatly complicate the multiphase systems. Spatial and temporal instability develops from field tests over decades, such as flow channelling through relatively small flow zones^17^. The spatial instability of two-phase flow in single fractures and fracture networks has been extensively investigated, experimentally ^10, 37–40^ and numerically^8, 41–44^, and attributed to the heterogeneity of fracture permeability and aperture. Despite these studies, the interfacial dynamics in rough fractures remain poorly understood because the widely used macroscopic mathematical models, based on solving the continuum Darcy’s law, neglect non-equilibrium and locally dynamic effects when the bulk flow is under laminar conditions. In addition, standard core-scale or field-scale models are often developed from empirical relationships using parameters that are easily measured in core-plug laboratory experiments^45^. While this phenomenological approach gives reasonable results for simple systems, it provides no means for understanding the local instability at the micron scale. The typical Haines jump shows a sudden increase in the interfacial velocity and a drop in the capillary pressure when the non-wetting phase (e.g., air, oil, mercury) passes from a pore neck into a wider pore body, displacing the wetting phase (e.g., water)^46^; the Haines jump is considered one of the intricate characteristic features of two-phase flow at the pore scale, along with snap-off, piston-like displacement, corner flow, ganglion dynamics and film swelling^47–49^. Discovered first more than 80 years ago in porous media^50^, the Haines jump has only recently attracted attention with the development of micron-scale visualization techniques^46, 51–59^. Using high-speed micro-CT at synchrotron facilities, Berg *et al*.^46^ presented their groundbreaking study on the phenomena in a 3D heterogeneous sandstone core. Real-time 3D imaging showed that the interfacial Haines jump is a type of cooperative pore-filling event, i.e., involving many individual pores and phase distributions, instead of one local pore and throat. Bultreys *et al*.^51^ reported Haines jumps in a Bentheimer sandstone core observed by a custom-built laboratory-based micro-CT scanner, giving more emphasis to the technological breakthrough. More importantly, whether the local Haines jump at the pore scale would average out for a bulk system remains debatable; some reports on porous media have shown its significant influences on the macroscale behaviour^46, 52–54^. In fractured media, this interfacial phenomenon has not been investigated.

In this study, experiments on the micron-scale Haines jump events of air-water flow in single fractures are conducted under drainage conditions. Six one-dimensional fracture models are investigated with varying *D* values from 1.0 to 1.5, representing variability in surface geometry from perfect flat to a certain roughness. The fracture model is first saturated with ink-dyed water and drained by air under a constant flow rate and atmospheric conditions. During the drainage experiments, high-resolution time-lapse images of the air-water interface and local aperture are obtained. The interfacial velocity is then calculated based on the distance travelled and corresponding time between two sequential images, and its dependency on the local aperture variations is discussed.

## Results

### Interfacial dynamics: Observation of Haines jump

**Figure 1:**
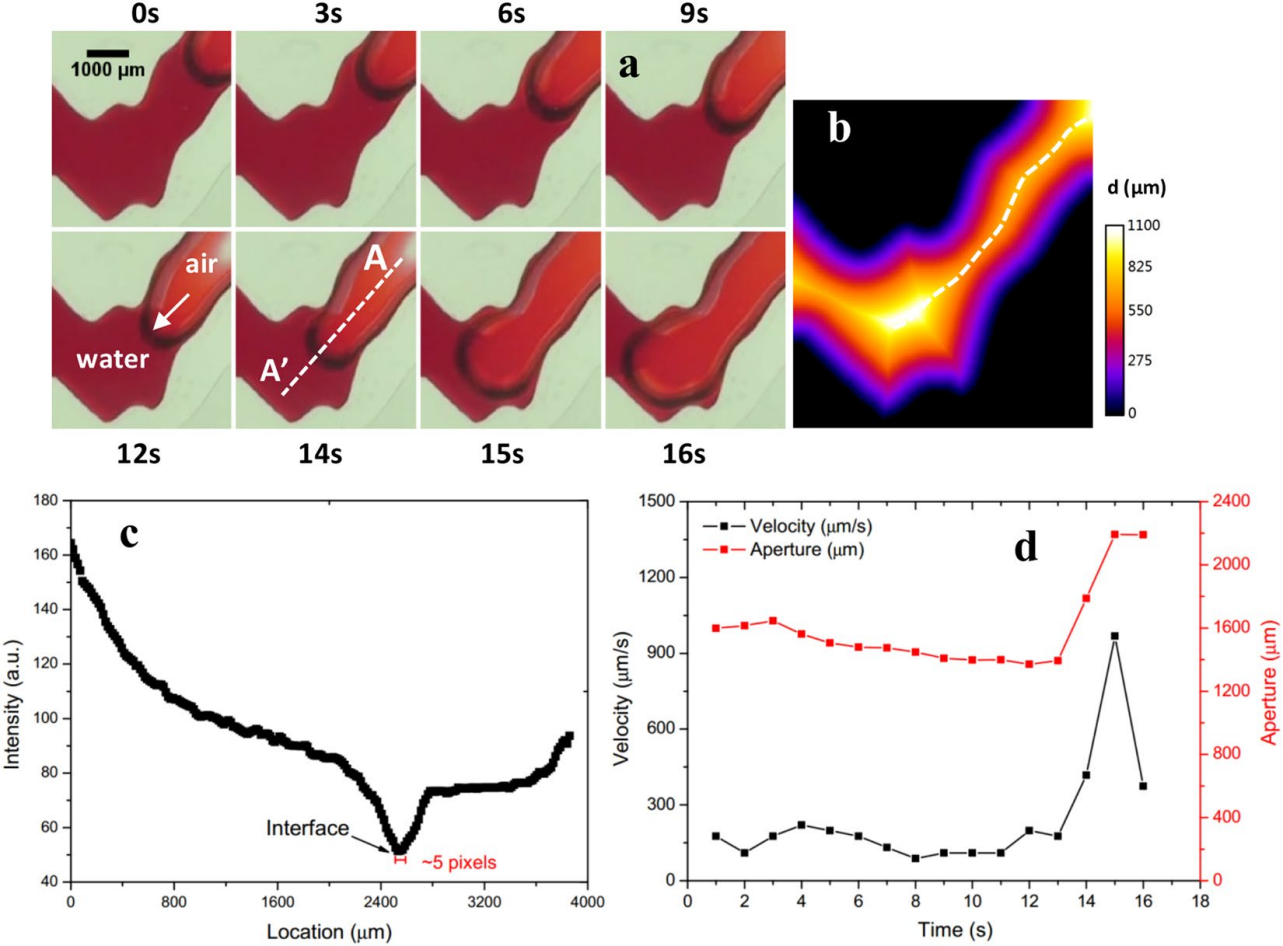
(**a**) Sample images demonstrating temporal Haines jump during air drainage. (**b**) Distance map of pixels within the fracture domain to the two impervious walls. The white dotted line indicates the maximum distance to the two boundaries, which is regarded as the flow-pathway centre. (**c**) Intensity profile along transect A-A’ showing reduced value at air-water interface, which is used for unambiguous interface discernment. (**d**) Temporal changes in interfacial velocity and width of aperture through which menisci flows. The white arrow in (**a**) indicates the air-water interface.

Haines jumps were observed in the fractures with rough surface during the air-water drainage experiments. We first present the local observations from the fracture model of *D* = 1.4 with a spatial resolution of 16 µm/pixel. Details of the aperture geometry for D = 1.4 and four other micromodels with D = 1.0 to 1.5 can be found in Figures S1 and S2 in the supporting material and in Ju *et al*.^36^. Figure 1 shows the local Haines jump that occurs when the air-water interface moves through a narrow “throat” to a large “body”. To quantify the aperture variation at local distance map of pixels within the fracture domain to the impervious walls was obtained by a Local Thickness Plugin in ImageJ^60, 61^, the fracture centre (largest distance to the impervious walls as shown by the white dotted line in Fig. 1b) can then be discerned and the local normal aperture is calculated. A sharp change in the intensity along the A-A’ profile in Fig. 1a is used to unambiguously signal the moving air-water interface as shown in Fig. 1c. As a result, the velocity of the air-water interface movement can be accurately calculated using two consecutive images. The same drainage experiment was conducted for each of the six fracture micromodels, and the interfacial dynamics were studied as a function of local aperture variability.

As shown in Fig. 1a,d, during the first 13 s, the menisci flows through the throat at a velocity of ~150 µm/s, which is less than the 208 µm/s bulk drainage velocity. When the menisci enters the large aperture “body” at 14 and 15 s, a sharp increase in the velocity to 418 and 964 µm/s is observed. The highest velocity jump is more than 6 times the average velocity in the throat and approximately 5 times the bulk drainage velocity. This velocity jump, as well as the interfacial dynamics of the two-phase air-water flow, can be attributed to the local aperture variations as a good correlation between the aperture and interface velocity is observed (see Fig. 1d).

The micron-scale interfacial dynamics is further investigated, as a function of local aperture variability, for three representative geometries: (1) in-phase roughness, where the longitudinal drainage-pathway length is close to the linear distance between inlet and outlet, while the transversal aperture varies spatially; (2) out-of-phase tortuosity, where the longitudinal drainage-pathway length is larger than the linear distance between two ends while the transversal aperture is relatively constant, expected at the inflection zones; and (3) a combination of these two phases. Similar definition on the above aperture geometry can be referred to Waite *et al*.^41^. We believe these typically sequential geometries may fundamentally affect the interfacial dynamics within a fracture network.

### In-phase roughness

**Figure 2:**
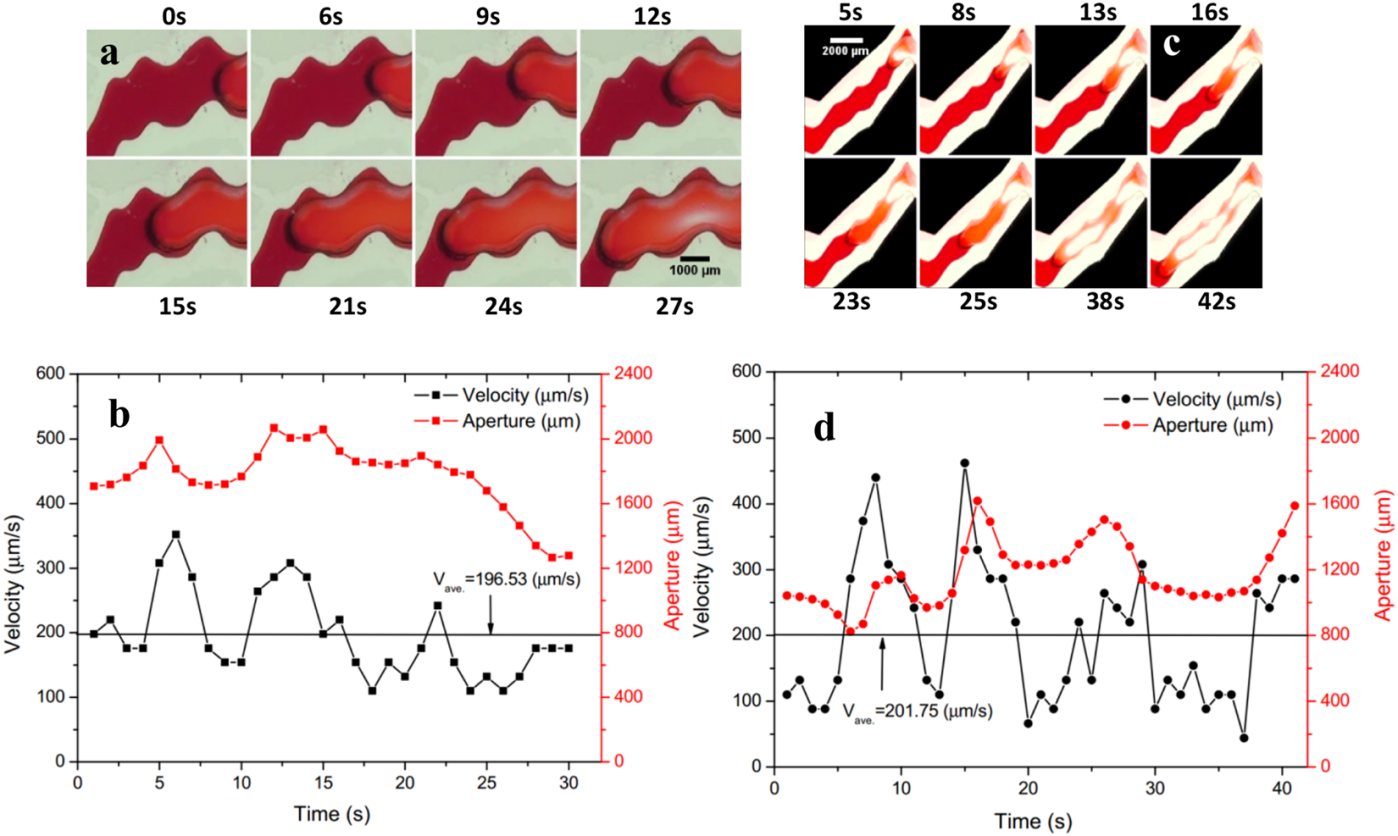
Time-lapse images showing interfacial dynamics in typical aperture geometries: (**a**) In-phase #1 and (**c**) In-phase #2. Temporal changes in the interfacial velocity and corresponding aperture width for In-phase roughness #1 (**b**) and In-phase roughness #2 (**d**).

The interfacial Haines jumps in two channel segments featuring in-phase roughness, labelled In-phases #1 and #2, are shown in Fig. 2a–d, respectively. For in-phase #1, it is apparent that the aperture variations yield velocity variations when the air-water interface passes through the segment of the fracture. The interface velocity decreases at low-aperture locations at 4, 10, and 20 s, and increases at larger-aperture ones at 6, 11, and 22 s. The maximum jump velocity is 352 µm/s at 6 s, 1.80 times the local average velocity (196.53 µm/s). The interface velocity becomes more stable after 20 s when the air-water interface passes through fracture aperture with no small-scale variability.

Similarly, for in-phase #2, the interface velocity exhibits temporal variations that correspond to aperture variations. The maximum jump velocity is 462 µm/s at 15 s, 2.30 times the average velocity of 201.75 µm/s. The deviation of velocity over the recorded 42 s induced by the aperture deviation of 194.42 µm is 105.27 µm/s and is higher than 68.21 µm/s induced by a smaller aperture deviation of 116.43 µm for In-phase #1. More detailed information on the aperture variations can be found in the Supporting Material.

### Out-of-phase tortuosity

**Figure 3:**
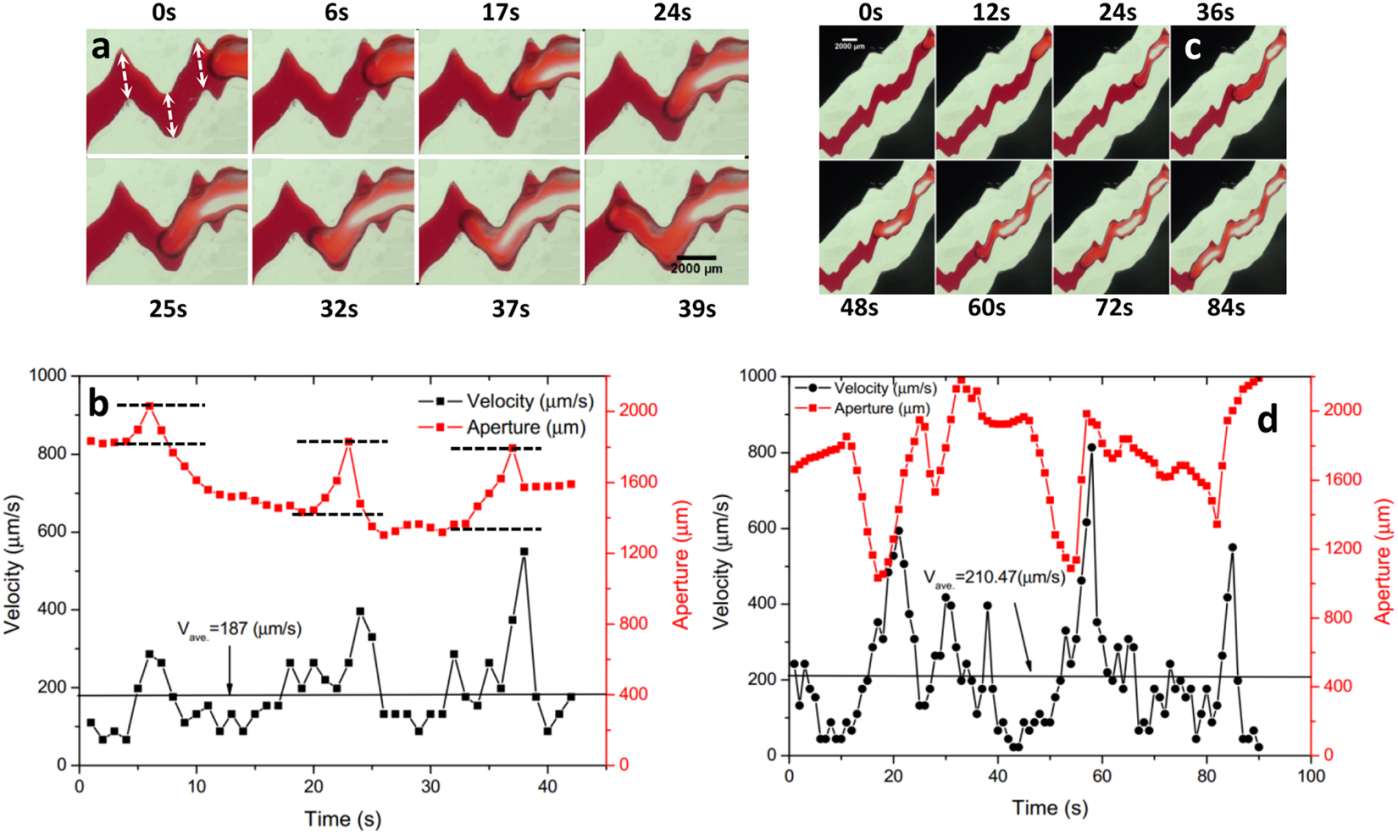
Time-lapse images showing interfacial dynamics in typical aperture geometries: (**a**) Out-of-phase #1 and (**c**) Out-of-phase #2. Temporal changes in interfacial velocity and corresponding aperture width for Out-phase #1 (**b**) and Out-phase #2 (**d**). The white arrows in (**a**) mark the three inflection zones in the “V”-shaped channel and the black dotted lines in (**b**) bound the aperture increases.

In contrast to the above two cases of in-phase roughness, we selected two channel segments with tortuous flow channel (out-of-phase #1 and #2 in Fig. 3) to investigate the corresponding interfacial jumps. As shown in Fig. 3a,b for out-of-phase #1, the Haines jump occurs at the three inflection zones of the “V”-shaped channel (marked by the white arrows in Fig. 3a) where the aperture increases, while the meniscus velocity maintains small but relatively constant values along the relatively flat channel. The jump velocity increases from 286 to 396 and to 550 µm/s as the menisci migrate through the three inflection zones, in correspondence with the increase in fracture aperture from 202.30, to 380.92 and to 452.45 µm (between black dotted lines in Fig. 3b). The local interfacial jump appears to be indirectly affected by the tortuosity of the flow channel, depending on the local variations of fracture aperture at the inflection zones. As a result, the aperture variations play a dominant role in the interfacial dynamics.

For out-of-phase #2, the meniscus velocity becomes unstable in time with larger jumps (see Fig. 3c,d). The maximum three jump velocities are 594, 814, and 550 µm/s that occur at 21, 58, and 85 s when the air-water interface passes through the fracture aperture of 1430, 1936, and 2001 µm, respectively. These larger velocity jumps occur at the sharply widened channel with higher temporal frequency for local small velocity variations when compared to out-of-phase #1. The observed variations in the interfacial velocity can be attributed to the spatial variability in fracture aperture.

### Dual-phase combination

**Figure 4:**
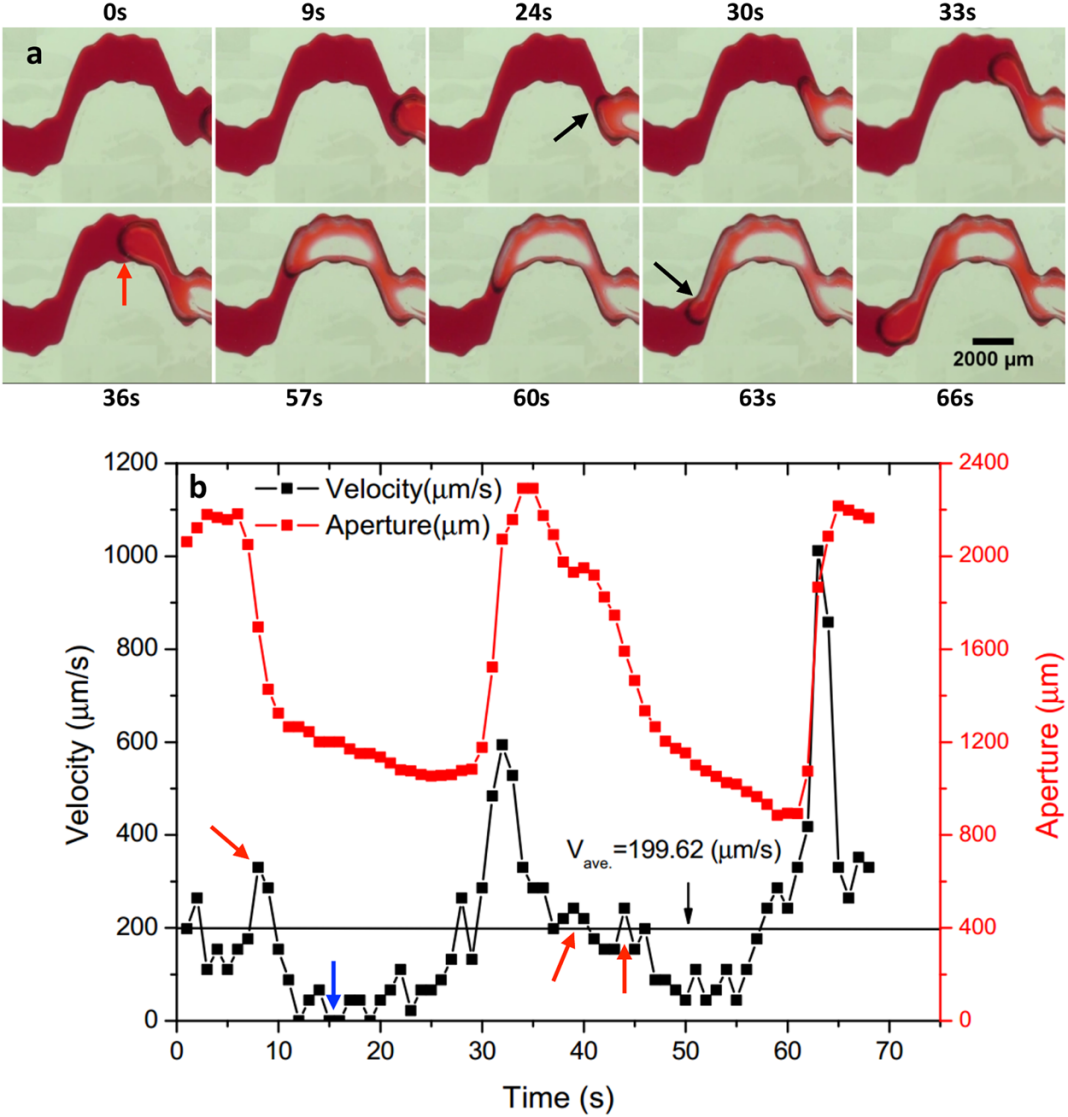
(**a**) Time-lapse images showing interfacial dynamics in the selected aperture geometry: Dual-phase combination (Dual-combo for short). (**b**) Temporal changes in interfacial velocity and corresponding aperture width. The black arrows in (**a**) indicate the two narrow “throats” at the inflection zones, while the red arrow indicates the wide “body”. The red arrows in (**b**) mark the smaller velocity increase at the jump intervals, while the blue arrow indicates the zero velocity as the interface invades the narrow throats for pressure build-up.

A dual-phase combination (Dual-combo for short) of fracture aperture was used to show its effect on the interfacial dynamics (see Fig. 4). In this case, a tortuous flow channel (out-of-phase tortuosity) was embedded into a local channel with aperture variability (in-phase roughness), leading to more significant aperture variations. The aperture ratios between the wide body (red arrow in Fig. 4a) and the two narrow throats at the inflection zones (black arrows) are 2.15 and 2.54. These sharp changes in aperture correspondingly result in the maximum Haines jump velocity of 594 and 1012 µm/s. Some smaller variations in interfacial velocity induced by the smaller aperture variations are also observed (see the red arrows in Fig. 4b). Note that nearly zero velocity occurs occasionally between 10 and 20 s as the menisci enter the narrow throats (see the second and third images in Fig. 4a and the blue arrow in Fig. 4b), where viscous pressure build-up is required for the increased capillary entry pressure. Again, the jump velocity and velocity variations show their strong dependence on largest and smallest aperture as well as smaller aperture variability along the fracture channel.

It is noted that the maximum jump velocities for the out-of-phase tortuosity cases in Fig. 3 and the dual-phase combination case in Fig. 4 are higher than those in the in-phase roughness cases in Fig. 2. Meanwhile, for the in-phase roughness, the maximum velocity is very close to the maximum fracture aperture, while in the out-of-phase tortuosity cases, the maximum velocity occurs slightly later than the maximum fracture aperture, rather coinciding with the largest gradient in the aperture. This may be attributed to the different throat travel distances for the invading interface. The longer throat travel distance for the out-of-phase tortuosity and dual-phase combination cases yields more elastic energy accumulation by a higher capillary pressure in the aperture ‘throat’. When the interface menisci enter a large aperture ‘pore’, the accumulative elastic energy releases more kinetic energy, resulting in a higher jump velocity at the zone with sharply reduced capillary pressure, where the largest gradient in aperture is. Similar observations have been reported by Berg *et al*.^46^ and Armstrong *et al*.^52^.

### Effect of fractal dimension

**Figure 5:**
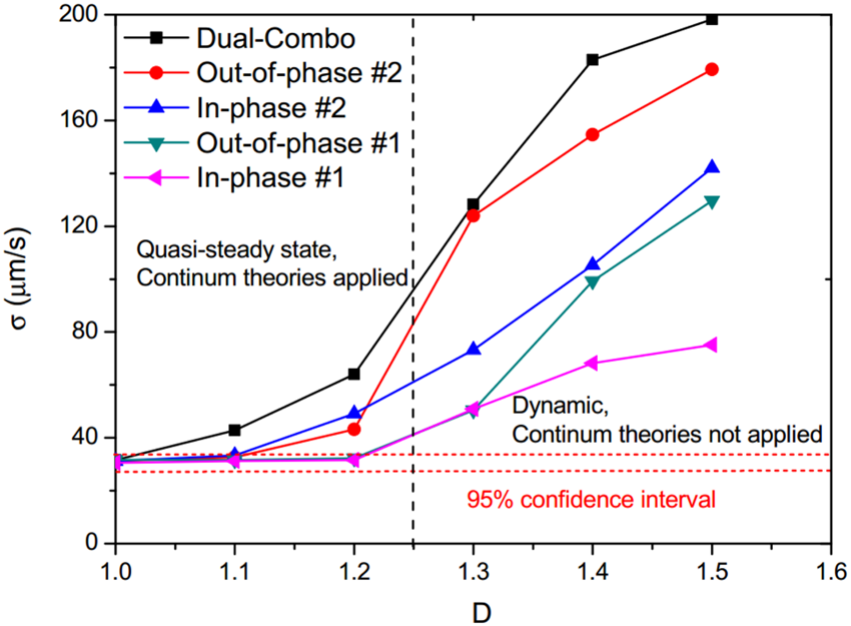
Correlation of the standard deviation (*σ*) of velocity variations and fracture *D* values in the five selected fracture segments.

We finally present the effect of parameter *D* on the interfacial phenomena in the five selected channel segments. The geometries of the selected fracture segments for varying *D* values can be found in Figures S3–S7 of the Supporting Material. The standard deviation (*σ*) of the velocity variations was calculated for each segment in each fracture micromodel. More details of their transient velocity can also be found in Figures S3–S7 in the Supporting Material. Figure 5 shows the correlation between *σ* and *D* for the five channel segments in six fracture models. For *D* = 1.0, the meniscus velocity is relatively constant at 208 ± 31 µm/s, and close to the bulk drainage velocity. The very small velocity variations may be primarily induced by errors in interface discernment, as the interface covers ~5 pixels in width shown by the narrow band with reduced intensity (see Fig. 1c). This high accuracy of velocity calculation for the perfectly flat model helps ensure that the measurement errors are negligible when compared to velocity variations induced by variable aperture. The velocity variations fall within the 95% confidence interval of *D* = 1.0 for most of the selected segments in the *D* = 1.1 case and two segments in the *D* = 1.2 case. For all these scenarios, the small aperture variability can only produce small temporal variations in the interfacial velocity, and the air-water flow can be considered as quasi-steady. For *D* > 1.2, temporal Haines jumps occur when the air-water interface moves along the fracture channel as long as surface roughness in space are sufficiently large. In this case, the location of the air-water interface is temporally unstable and the continuum theory may not be applicable.

## Discussion

Natural fractures have significant variabilities in their surface roughness and two-phase flow in these fractures exhibits temporal unstable interfacial dynamics. This study provides direct observations of the interfacial dynamics of air-water flow in single one-dimensional fractures with varying surface roughness. These observations may have the following implications for multiphase fluid flow in naturally and artificially developed fracture media: (1) an interfacial Haines jump may develop within a fracture segment with large aperture variations locally, with the highest jump velocity observed ~5 times the bulk drainage velocity; (2) the dynamic interface velocity results in more residual water in narrow “throats” than in wide “bodies” (see Figs 2–4), which may explain the origin of non-uniform phase distribution and considerable variability in the lumped relative permeability.

The observed Haines jump can be attributed to the local aperture-dependent competition between capillary and viscous forces. In a Haines jump, the elastic energy initially contained in the liquid–liquid menisci at “throat” by higher capillary force is converted into kinetic energy, with substantial inertial contributions when the menisci move to the “body” with sharply reduced capillary force^46, 55^, and finally dissipated. A comparison of the phenomena in a single fracture and a well-connected pore network may aid in understanding the interaction between this locally dynamic process and the media connectivity. For instance, in 2D homogeneous micromodels constructed from glass beads with similar pores (1.6−2.0 mm diameter) and pore-throat sizes (1.0 mm diameter) at a coordination number of 4, Moebius and Or^55^ observed interfacial velocities exceeding 50 times the mean front velocity, with the neighbouring throats reversing (liquid redistribution). By using a micromodel with a hexagonal pore-throat geometry at a coordination number of 6, Armstrong *et al*.^52, 53^ obtained a maximum interfacial velocity approximately three orders of magnitude greater than the average mean pore velocity. In addition, they observed a cooperative drainage event, i.e., the local capillary pressure differences extend over multiple pores and directly affect the fluid topology and meniscus dynamics, indicating the effect of the interfacial phenomena at a local pore/throat on the phase distribution at the pore-network scale. This cooperative drainage event was further verified by Zacharoudioua and Boeka^59^ through lattice Boltzmann simulations. Compared to the relatively small increase in the jump velocity in the single rough fracture (5 times) in this study, we may conclude that these interfacial dynamics exhibit a nonlinear dependence on the flow path connectivity. Additional experiments were conducted by Moebius and Or^56^ with the aim to establish a quantitative link between the macroscopic boundary conditions (bulk displacement rate) and local invasion dynamics. The results showed that the distribution of the invasion event volumes was only mildly dependent on the macroscopic drainage rate and was practically independent of the resulting fraction of the residual phase entrapped behind the drainage front in the absence of gravity. Finally, in 2014, a homogeneous pore-throat network model was presented^57^ for quantifying the interfacial dynamics and interactions along the fluid displacement front. Pore throat sizes from 0.2–0.7 mm with two different standard deviations, 0.2 and 0.4 mm, were investigated. The modelling results showed that higher standard deviations of the pore throat size yielded larger interfacial jump velocities, corresponding to the exponential correlation between the velocity and aperture variations in this study. Moreover, our results present the interfacial dynamics within apertures of standard deviations ranging from 0.1 to 0.5 mm and thus give a more detailed understanding of the dependence of the interfacial dynamics on the aperture geometry of fractures.

**Figure 6:**
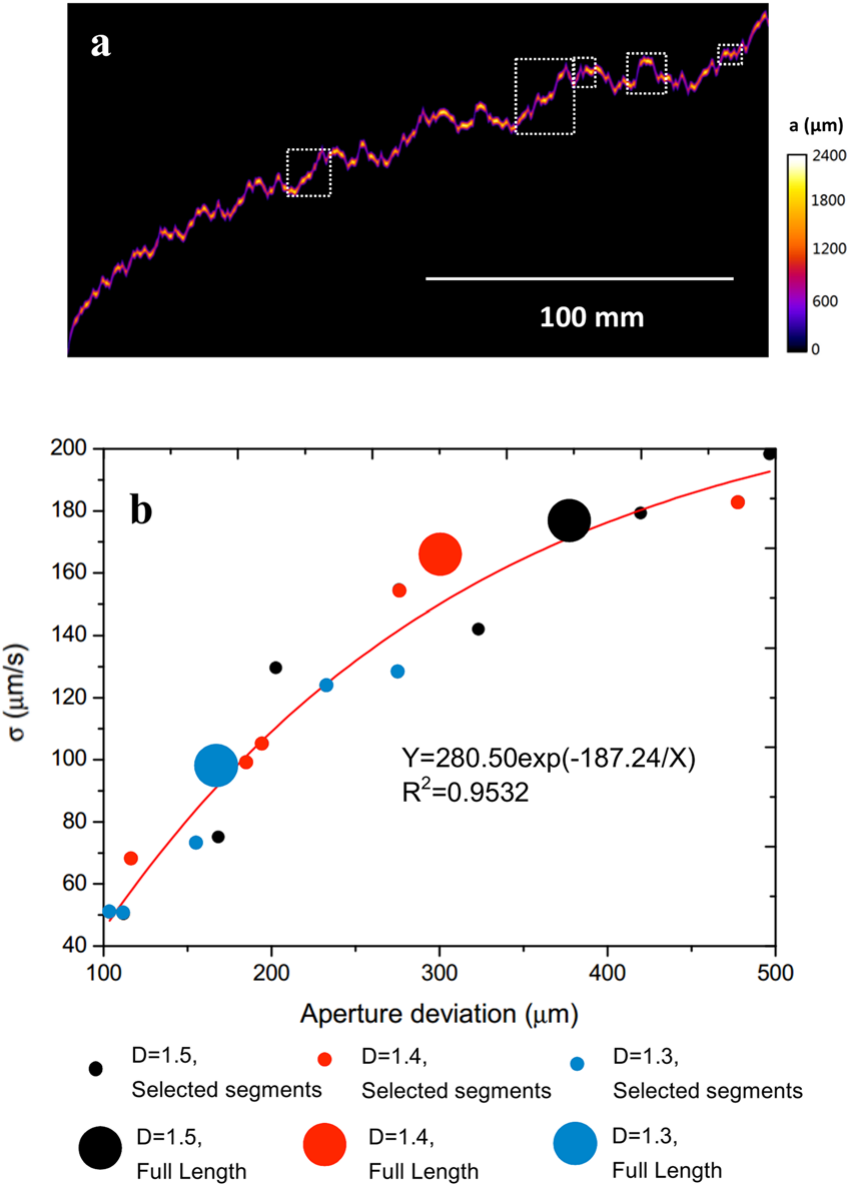
(**a**) Geometry of the single rough fracture at fractal dimension *D* = 1.4 in full length, with different colours (from purple to white) to show the aperture width. (**b**) Correlation of velocity variation (*σ*) and local aperture deviation in the five selected fracture segments and in full fracture channels for micromodels *D* > 1.2. The white dotted boxes in (**a**) mark the locations of the five segments selected for interpretation of interfacial dynamics in different sequential geometries.

We further statistically determined the correlation between the velocity and aperture deviations for the five selected segments at a length scale of ~100 µm and for the entire fracture channels at ~100 mm scale in fracture micromodels D > 1.2, in which the interfacial velocity cannot be predicted by continuum Darcy theory. Figure 6a shows the aperture geometry of the single fracture micromodel at fractal dimension *D* = 1.4 in full length, with different colours (from purple to white) to show the aperture width. Locations of the five selected segments are also shown by the white dotted boxes. Correlations of velocity variation (*σ*) and aperture deviation in the five selected fracture segments (small dots) and in the full fracture channels (large dots) for micromodels *D* > 1.2 are shown in Fig. 6b. Note that we focused on a full loop of the interfacial dynamics, including the Haines jump and velocity breakdown during pressure build-up in the sequential aperture variations, rather than a Haines jump only within a single throat-body sequence in porous media^52, 55^. It appears that the velocity variation for the five channel segments increases with the increase in aperture deviation in an exponential function. The expected highest velocity variation is 270 µm/s for the air-water displacement under the fracture conditions. This behaviour is expected because of the interfacial tension between the invading air and displaced water. Note that the oil-water jump velocity is one order of magnitude higher than that for air-water in porous media; the air-water interfacial tension is 72 mN/m while oil-water interfacial tension is 21 mN/m^52, 55^. Also shown in Fig. 6b, the three data points at the ~100 mm length scale for the entire channel also follow the exponential correlation obtained from the ~100 μm length scale. This indicates that the proposed correlation is scale-invariant, at least for the interfacial dynamics observed in a single rough fracture with sufficient aperture variations. Beyond singe fracture, we expect that such interfacial dynamics of two-phase flow will be more significant in 2D and 3D fracture networks with higher aperture variations.

## Methods

Drainage experiments in rough fractures were conducted to investigate the interfacial dynamics of air-water flow at micron scale. The fractures were created using a fracture model with varying fractal characteristics (i.e., *D*). Before each experiment, the same experimental procedure involving dyed-water preparation and saturation measurement was followed. During the experiment, time-lapse images of the air-water interface and fracture aperture were obtained using an imaging system. These images were used to calculate the menisci displacement velocity.

### Fracture micromodels

Six fracture micromodels were created following Ju *et al*.^36^. For each micromodel, the trajectory of fracture half-aperture was generated from a Weierstrass–Mandelbrot fractal function:

1 $$W(t)=\sum _{n=-\infty }^{\infty }({1}-{e}^{i{b}^{n}t}){e}^{i{\varnothing }_{n}}/{b}^{{({2}-D)}^{n}},$$

where the constant *b* is a real number greater than 1.0, reflecting the deviation degree of a curve from a straight line, ${\varnothing }_{n}\,$represents an arbitrary phase angle, and *D* ∈ (1, 2). The fractal governing function, *C*(*t*), is the real part of *W*(*t*), such that

2 $$C(t)={Re}W(t)=\sum _{n=-\infty }^{\infty }({1}-{\rm{co}}{{\rm{s}}}^{{b}^{n}}t)/{b}^{{({2}-D)}^{n}}.$$

A photomask was converted from two identical *C*(*t*) curves to form fracture aperture from the two opposite surfaces (i.e., fracture walls). The six fracture micromodels were generated, with *D* = 1.0, 1.1, 1.2, 1.3, 1.4, and 1.5, respectively, and fabricated on a polymethyl methacrylate (PMMA) plate using a well-controlled laser cutter. These micromodels were then sandwiched and bonded between two flat PMMA plates. The aperture for the flat tube model (*D* = 1.0) was designed as 2.0 mm, while for D > 1.0, the average normal aperture, determined by the two-dimensional area of the fracture domain and the boundary wall length, was set as 2.0 mm. Details on the fracture micromodels are shown in Table 1 and in the Supporting Materials. We ensured no leakage during the drainage experiments by tracking the dyed-water within the fracture and by confirming a good match between the injected and outflow water volumes. We focused on the air-water flow phenomena in the fracture channels bounded by impervious fracture walls. The absolute permeabilities of the fracture channels were measured under ambient pressure and temperature.

**Table 1:** Characteristics of the rough fracture models.

D	1.0	1.1	1.2	1.3	1.4	1.5
^**a**^ **Length (cm)**	21.77	22.55	23.73	26.44	32.13	40.04
**Average aperture (cm)**	0.20	0.20	0.20	0.2	0.20	0.20
**Depth (cm)**	0.4					
**Area (cm** ^**2**^ **)**	4.35	4.51	4.75	5.29	6.43	8.01
^**a**^ **Permeability (×10** ^**−7**^ ** m** ^**2**^ **)**	3.12	2.21	1.32	1.25	0.84	0.73

^a^Ju *et al*.^36^.

### Experimental setup

**Figure 7:**
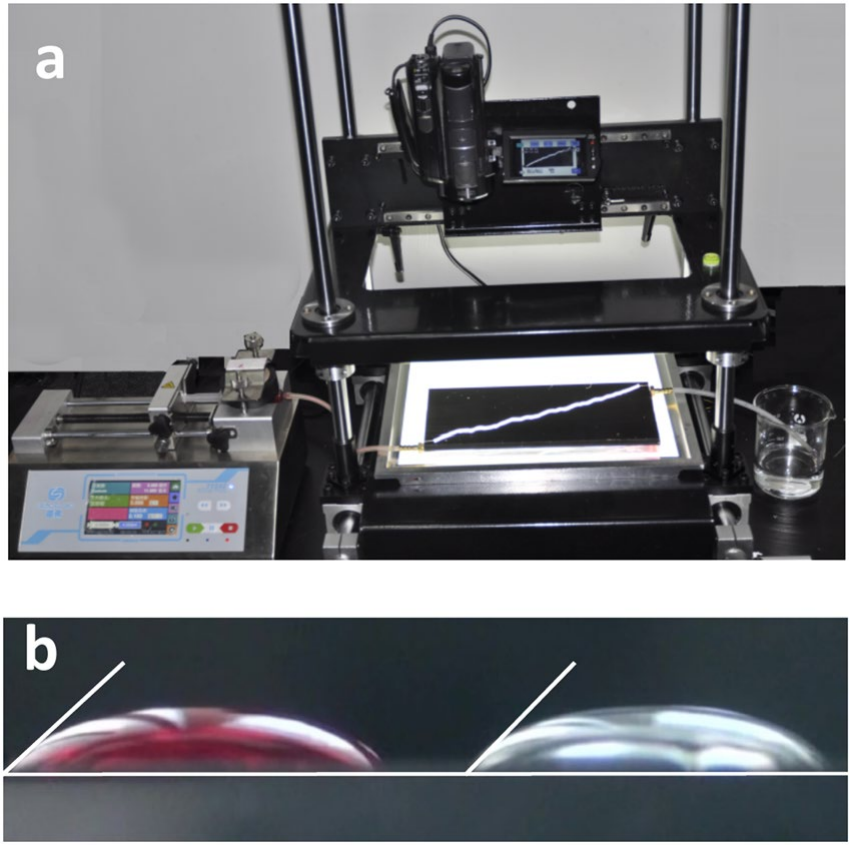
(**a**) Experimental setup. (**b**) Contact angle measurements of water with and without dye on PMMA surface.

An experimental setup with an imaging system was used for each drainage experiment (Fig. 7a). The transparent fracture micromodel was placed horizontally on a white-light-emitting board to enhance the imaging contrast. The model inlet was connected to a well-controlled syringe pump for water injection and withdrawal. The outlet was in contact with atmosphere under room conditions. A Sony HDR-SR12E charge-coupled-device (CCD) camera (Sony Corp., Tokyo, Japan) was assembled above the fracture model against a scope stage adjustable in three directions. During the experiment, the camera height was fixed with the stage, while the horizontal position was adjusted for optimal observation of the mobile air-water interface.

### Experimental procedures

Before the drainage experiment, distilled water was dyed with red ink for superior phase discernment. This had little effect on the air-water contact angle on the PMMA surface as the contact angle measured was 44° and 47° before and after dying (see Fig. 7b), indicate strong water-wetness of the PMMA surface. It was also expected that the water dying had a little effect on the interfacial tension because of the low ink concentration used. The dyed-water was then injected into the fracture model at a low rate to establish fully water-saturated conditions, with no trapped air bubbles. Both the fluid (in its entirety) and the fracture model were kept at room temperature.

After the above preparatory steps were completed, the drainage experiment was conducted by withdrawing the syringe pump at a constant flow rate of 0.1 mL/min that corresponded to a velocity of 208 µm/s. The capillary number (*Ca*) was calculated as −7.14 (logarithmic value) using *Ca* = (*μ* × *v*)/(*σ* × cos*θ*), where *μ* (=1.79 × 10^−5^ pa. s) and *v* are the viscosity and Darcy velocity of air, respectively, and *σ*(=0.072 N/m) and *θ* are the interfacial tension and air-water contact angle, respectively^39^. The low *Ca* and viscosity ratio (logarithmic value: −1.75) indicates the displacement is dominated by capillary pressure, referring to the Log*Ca* − Log*M* stability diagram in Zhang *et al*.^62^. Thus, the variability in fracture aperture may have a significant effect on interfacial dynamics. A similar displacement rate was used to investigate the two-phase flow characteristics in the rough-walled fracture^39^.

During the drainage experiment, high-resolution time-lapse images of the aperture variations and air-water interfaces were recorded by the CCD camera, while the interface discernment and velocity calculation were conducted using ImageJ.

## Electronic supplementary material

Supplementary Information
