# Supplementary material for: Non-Darcy interfacial dynamics of air-water two-phase flow in rough fractures under drainage conditions
Source: Sci Rep. 2017 Jul 4;7:4570. doi: 10.1038/s41598-017-04819-x (PMC5496895; doi:10.1038/s41598-017-04819-x)
Supplement: Supplementary file 1 — Supplementary Information [file 41598_2017_4819_MOESM1_ESM.doc]

***Supporting information***

Non-Darcy interfacial dynamics of air-water two-phase flow in rough fractures under drainage conditions

**Chun Chang^1^, Yang Ju^*1,2^, Heping Xie^3^, Quanlin Zhou^4^, Feng Gao ^2^**

^1^ State Key Laboratory of Coal Resources and Safe Mining, China University of Mining & Technology at Beijing, D11 Xueyuan Road, Beijing 100083, P. R. China

^2^ State Key Laboratory for Geomechanics and Deep Underground Engineering, China University of Mining & Technology, No 1, University Avenue, Xuzhou 221006, P. R. China

^3^ Key Laboratory of Energy Engineering Safety and Mechanics on Disasters, The Ministry of Education, Sichuan University, Chengdu 610065, China

^4^ Energy Geosciences Division, Lawrence Berkeley National Laboratory, Berkeley, California, CA94720, USA

*** Corresponding author.** Phone: (+86) 10 62331490; fax: (+86) 10 62331253; e-mail: [juy@cumtb.edu.cn](mailto:juy@cumtb.edu.cn)

Scientific Reports

Number of pages: 8

Number of figures: 7

**Date prepared: Mar. 10**

**
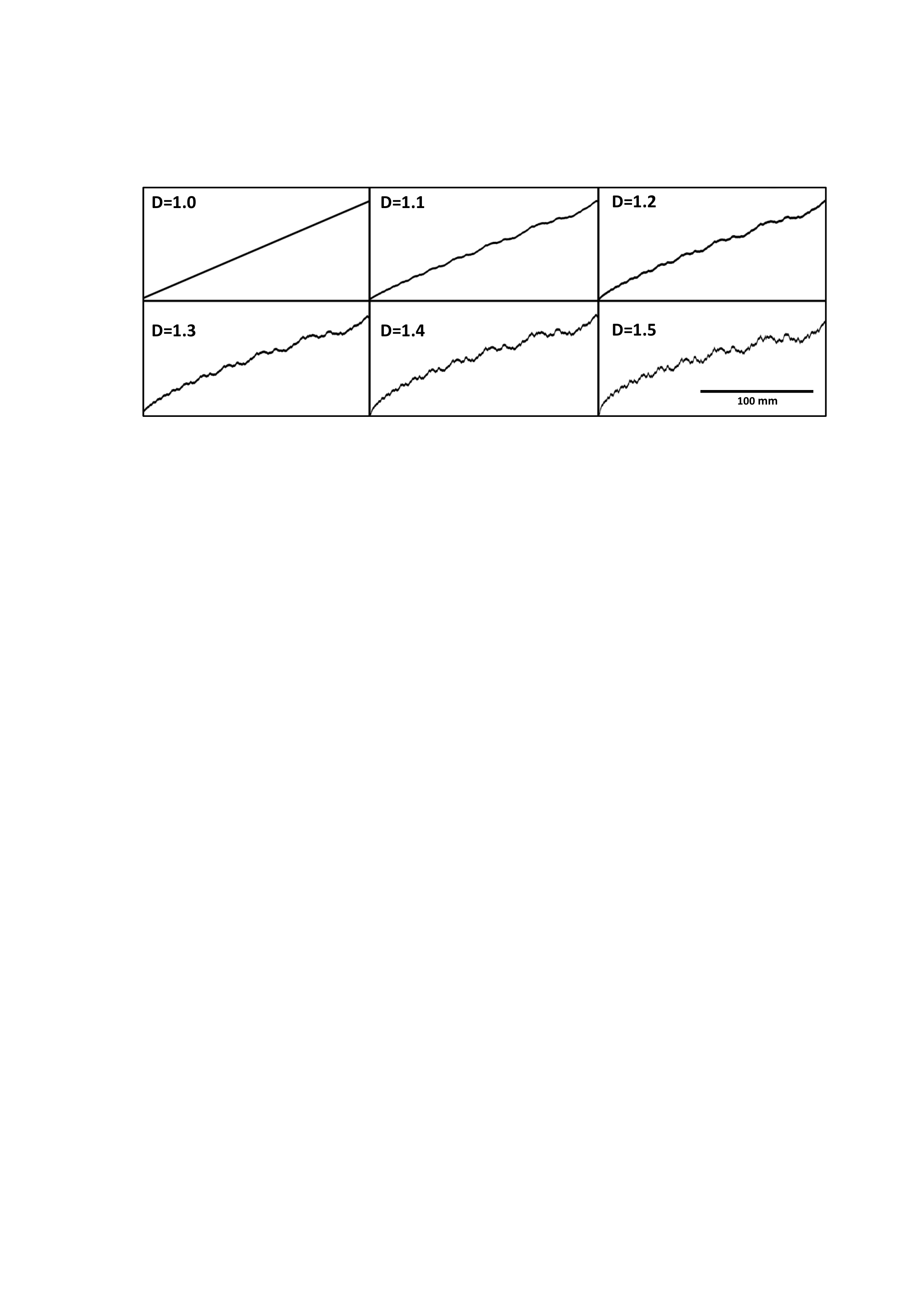
**

**Figure S1.** Schematic of the six rough fracture models with varying fractal dimensions.


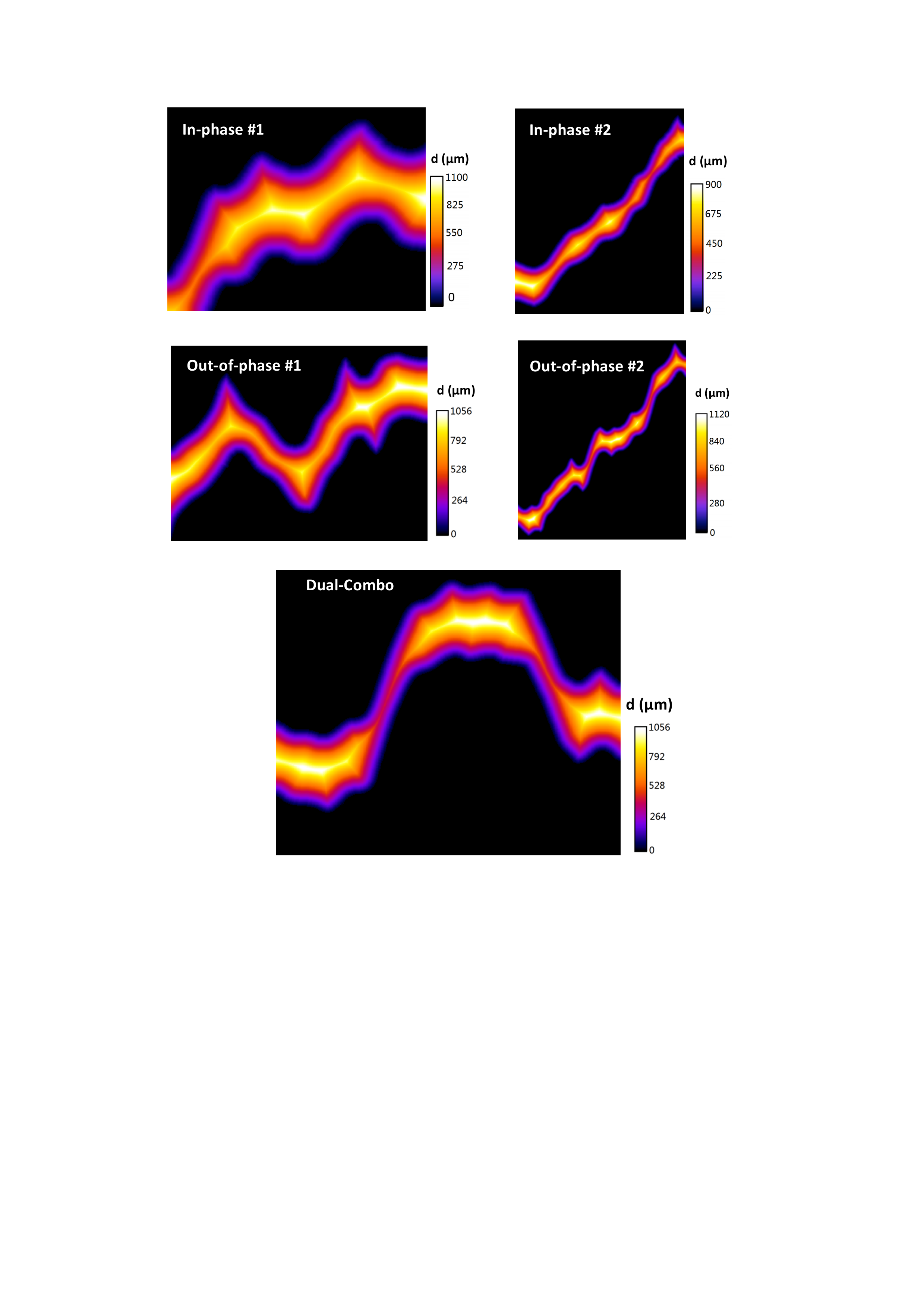


**Figure S2.** Distance maps for the selected segments in *D*=1.4, which is quantified by the Local thickness plugin in Imagej software.

**
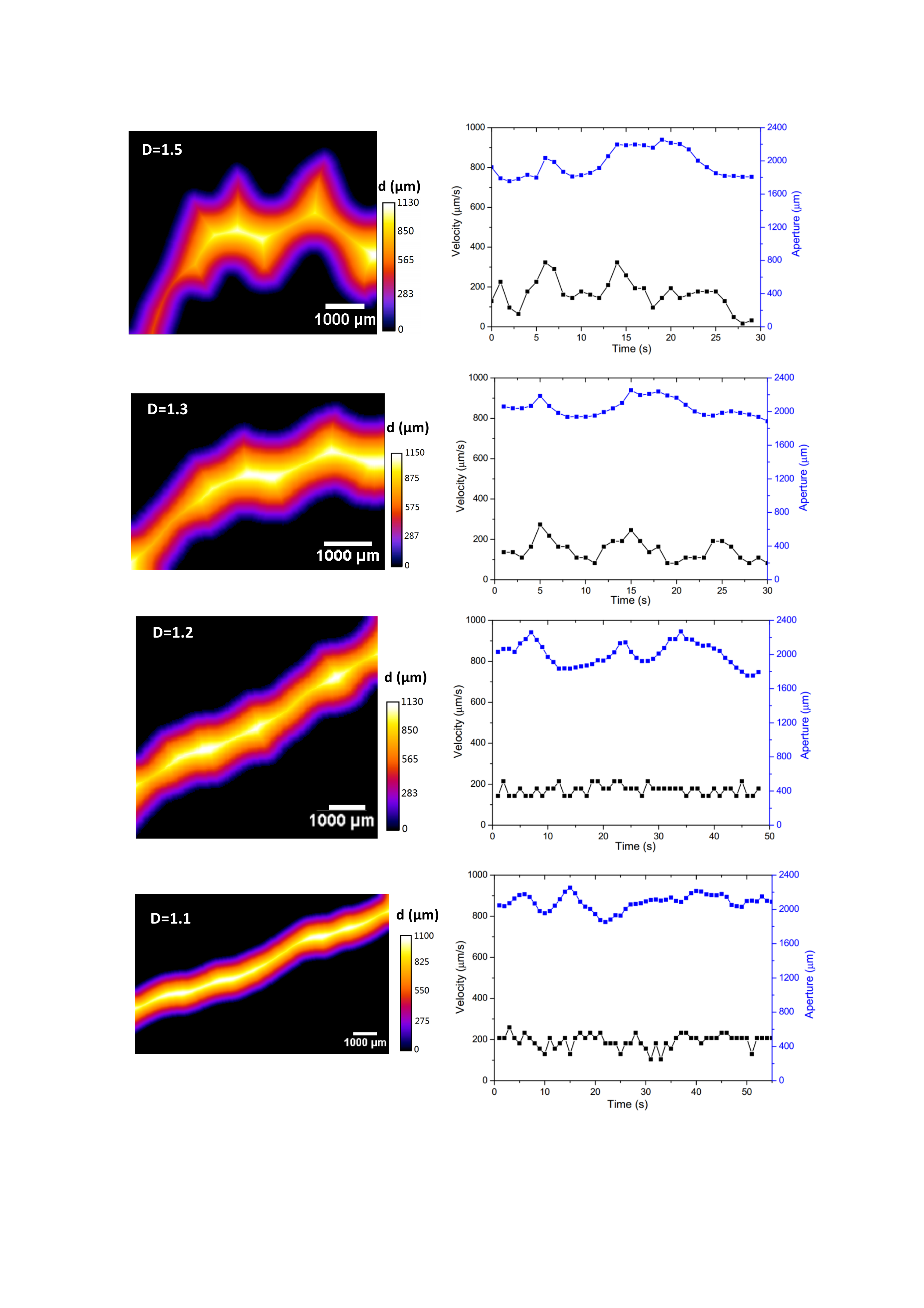
**

**Figure S3.** Distance maps (left panel) for In-phase #1 in fracture micromodels with varying *D* values and the corresponding interfacial velocities (right panel).

***
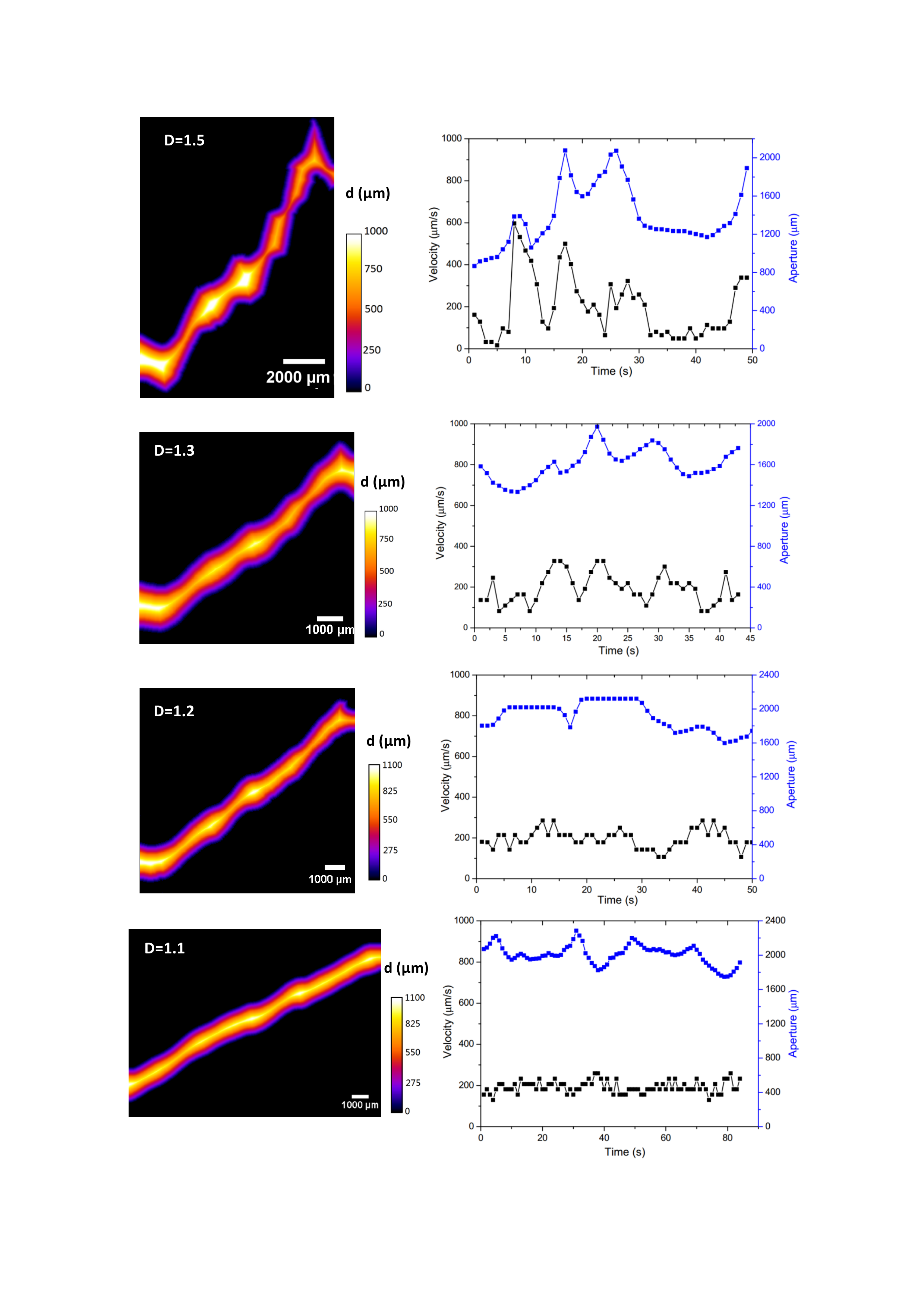
***

**Figure S4.** Distance maps (left panel) for In-phase #2 in fracture micromodels with varying *D* values and the corresponding interfacial velocities (right panel).

**
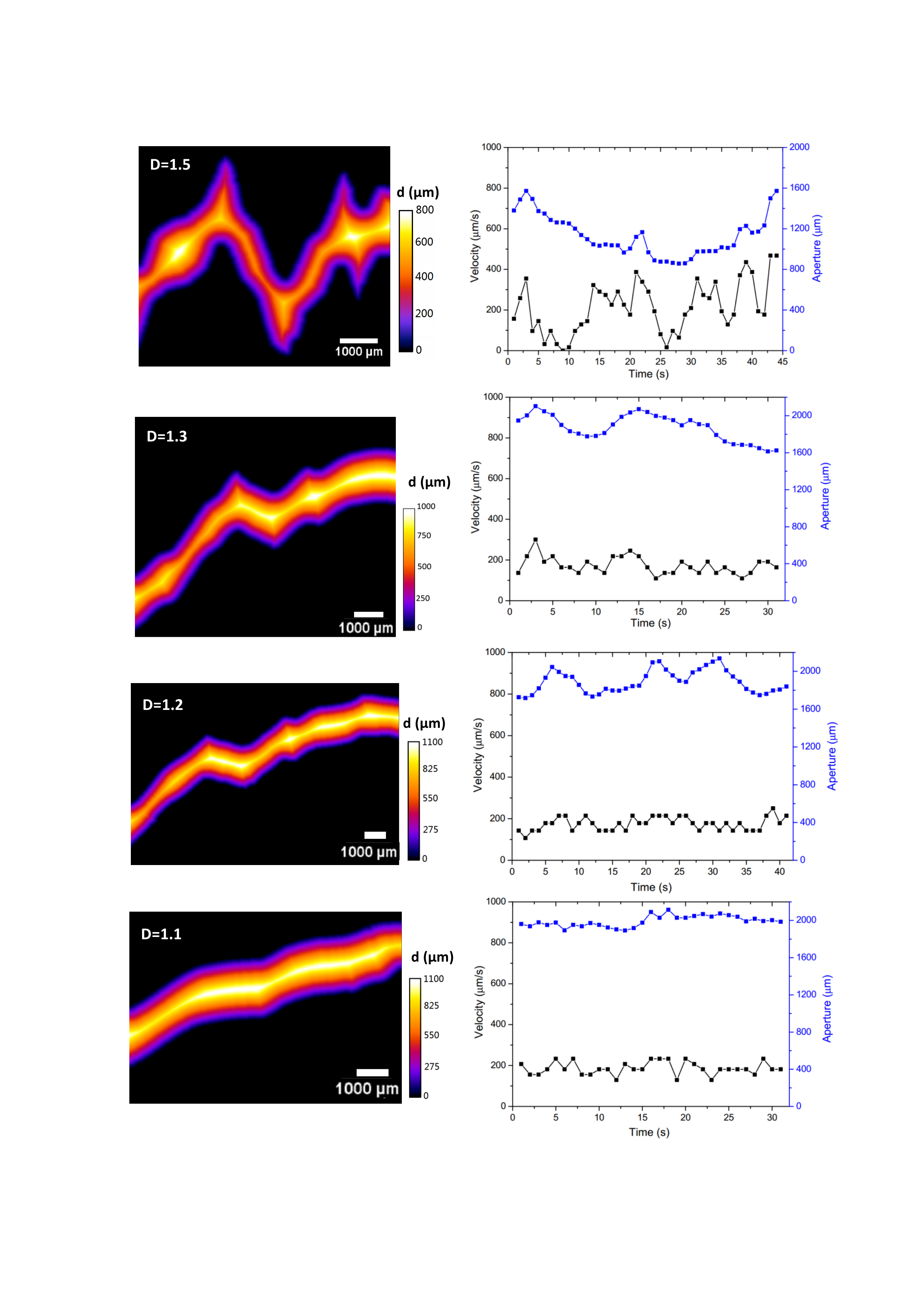
**

**Figure S5.** Distance maps (left panel) for Out-of-phase #1 in fracture models with varying *D* values and the corresponding interfacial velocities (right panel).

**
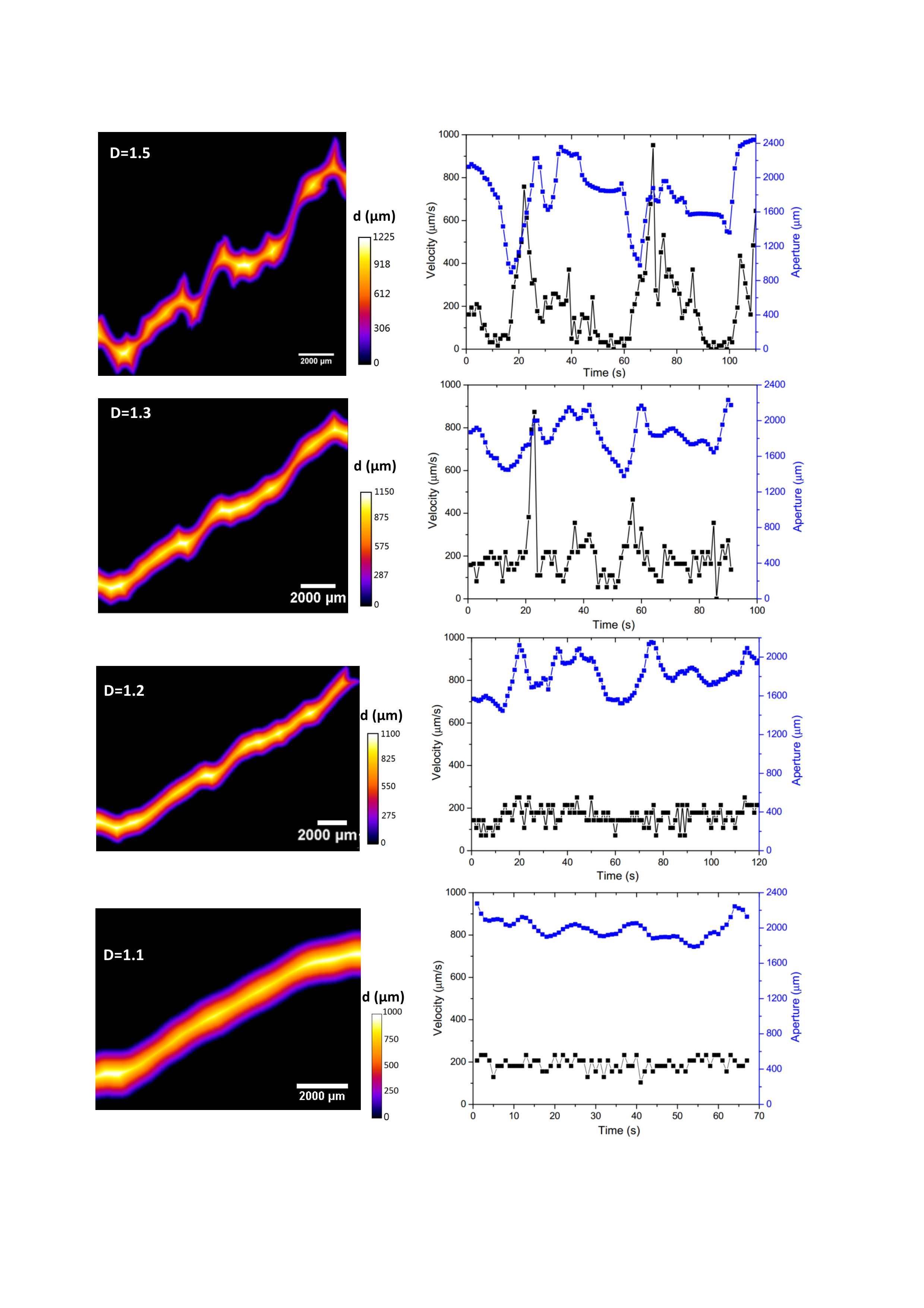
**

**Figure S6.** Distance maps (left panel) for Out-of-phase #2 in fracture micromodels with varying *D* values and the corresponding interfacial velocities (right panel).

***
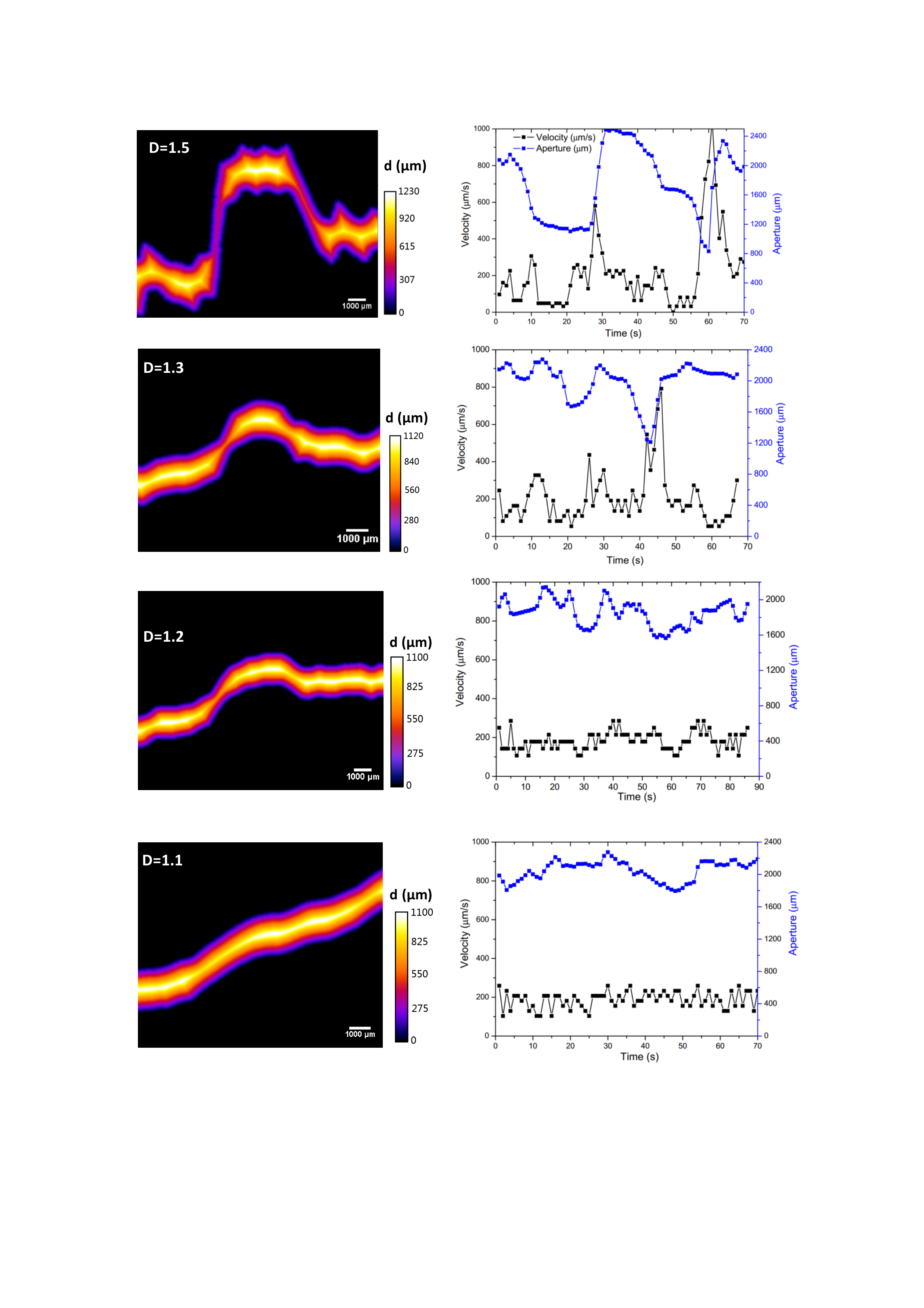
***

**Figure S7.** Distance maps (left panel) for case Dual-Combo in fracture micromodels with varying *D* values and the corresponding interfacial velocities (right panel).
